# Supplementary material for: Effects of Organic Acids on the Release of Fruity Esters in Water: An Insight at the Molecular Level
Source: Molecules. 2022 May 4;27(9):2942. doi: 10.3390/molecules27092942 (PMC9100015; doi:10.3390/molecules27092942)
Supplement: Supplementary file 1 [file molecules-27-02942-s001.zip › molecules-1695015-supplementary.pdf]

Table S1. Partition coefficient of six esters in different matrix (*k*×100)

Esters / Matrix	C2C3	2MeC3C2	C4C2	C2C4	3MeC4C2	C5C2
Water	1.30±0.00	3.91±0.11	2.51±0.13	2.14±0.20	8.88±0.02	4.67±0.15
1 g/L CA	1.13±0.02	3.60±0.13	2.23±0.04	1.97±0.03	9.09±0.04	3.34±0.11
2.5 g/L CA	1.23±0.00	3.44±0.08	2.55±0.02	1.96±0.12	9.79±0.04	3.70±0.15
5 g/L CA	1.27±0.02	3.61±0.00	2.62±0.03	2.15±0.11	10.99±0.06	4.30±0.17
1 g/L MA	1.23±0.00	3.86±0.01	2.32±0.06	2.15±0.08	8.45±0.03	3.96±0.02
2.5 g/L MA	1.25±0.02	3.90±0.09	2.39±0.14	2.31±0.11	10.28±0.10	4.28±0.01
5 g/L MA	1.28±0.02	3.45±0.15	2.41±0.05	2.40±0.06	11.26±010	4.60±0.00
1 g/L LA	1.07±0.03	3.10±0.11	2.77±0.04	2.01±0.08	8.37±0.03	3.97±0.03
2.5 g/L LA	1.17±0.02	3.16±0.11	3.05±0.05	1.96±0.06	8.20±0.02	3.86±0.02
5 g/L LA	1.18±0.00	3.00±0.04	2.84±0.02	1.75±0.07	7.98±0.05	3.69±0.02

Table S2. The interaction mode between citric acid and esters

Esters	Hydrogen bonds	Free CA	Steric hindrance	Ternary ring structure
C2C3	2	2	—	—
2MeC3C2	2	—	Y[1]	Y
C4C2	3	—	—	Y
C2C4	2	—	—	Y
3MeC4C2	2	—	Y	Y
C5C2	4	—	—	—

[1] The Y means the type of this interaction exists between esters and CA

Table S3. The interaction mode between lactic acid and esters

Esters	Hydrogen bonds	Free CA	Steric hindrance	Ternary ring structure
C2C3	1	1	—	—
2MeC3C2	2	—	—	Y
C4C2	—	—	—	—
C2C4	2	—	Y[1]	—
3MeC4C2	1	—	—	—
C5C2	2	—	—	—

[1] The Y means the type of this interaction exists between esters and CA

Table S4. The interaction mode between lactic acid and esters

Esters	Hydrogen bonds	Free CA	Steric hindrance	Ternary ring structure
C2C3	2	2	—	—
2MeC3C2	3	—	Y	Y
C4C2	2	1	—	—
C2C4	1	—	—	—
3MeC4C2	2	—	Y	—
C5C2	2	—	—	Y

[1] The Y means the type of this interaction exists between esters and CA
